# Perfectionism and Burnout During the COVID-19 Crisis: A Two-Wave Cross-Lagged Study

**DOI:** 10.3389/fpsyg.2020.631994

**Published:** 2021-02-01

**Authors:** Paola Spagnoli, Carmela Buono, Liliya Scafuri Kovalchuk, Gennaro Cordasco, Anna Esposito

**Affiliations:** Department of Psychology, University of Campania “Luigi Vanvitelli”, Caserta, Italy

**Keywords:** perfectionism, burnout, cross-lagged panel study, path analysis, Burnout Assessment Tool

## Abstract

The current study aims at examining the relationship between the perfectionism two-factor model (i.e., concerns and strivings) and burnout dimensions measured by using the BAT (Burnout Assessment Tool) through a longitudinal study. A two-wave cross-lagged study was conducted using path analysis in SEM (Structural Equation Modeling) of 191 workers. Results confirmed the predictive role of perfectionistic concerns on the burnout dimensions, whereas perfectionistic strivings were not significantly related, suggesting that perfectionism should be monitored by employers and clinicians to prevent employee burnout. Limitations and future research directions are envisaged.

## Introduction

Perfectionism encompasses the tendency to set high personal standards and critically evaluate the self’s adequacy in reaching those standards (Slaney et al., 2001). Although perfectionism significantly influences various life domains (Stoeber and Stoeber, 2009), research on it has been primarily conducted in clinical and educational settings with few investigations being made in working environments. The existing studies indicate that perfectionism shares positive relationships with psychological strain (e.g., Mitchelson and Burns, 1998), burnout (e.g., Stoeber and Rennert, 2008), work engagement (e.g., Childs and Stoeber, 2010), and workaholism (e.g., Falco et al., 2017). A recent meta-analysis confirmed these associations (Harari et al., 2018). To our knowledge, current available findings on work-related perfectionism are cross-sectional, excluding the studies proposed by Childs and Stoeber (2012) and Flaxman et al. (2012), which are longitudinal. Thus, more longitudinal contributions are needed to clarify how perfectionism at the workplace can be predictive of employees’ stress and burnout.

In order to fill this gap, this study focuses on the longitudinal relationship between perfectionism and burnout, which is a multidimensional phenomenon reflected in “overwhelming exhaustion, feelings of cynicism and detachment from the job, and a sense of ineffectiveness and lack of accomplishment” (Maslach, 2003, p. 190). In addition, burnout constitutes “a psychosocial syndrome associated with motivational, performance, and psychological difficulties” (Hill and Curran, 2016, p. 269).

This study was conducted in Italy during the first lockdown due to the COVID-19 emergency when the majority of workers were working remotely, while those working on location were under enormous pressure from the emergency conditions and stressed by the perceived risk of being infected (i.e., nurses, doctors, police, supermarket clerks, etc.). This was a particularly unusual context, which adds value, given the circumstances to the present research aiming at providing robust evidence of the relationship between perfectionism and burnout at the workplace through a two-wave cross-lagged study.

### Dimensions of Perfectionism

Perfectionism is generally considered a multidimensional construct (Stoeber and Otto, 2006). Most of the scholars agree that perfectionism contains two underlying dimensions: perfectionistic concerns and perfectionistic strivings (Stoeber and Otto, 2006; Rice et al., 2014). Perfectionistic concerns are described as the fear of making mistakes and being overly critical of one’s performance. Perfectionistic strivings are characterized as holding high performance-related expectations for oneself. Previous research has suggested that varying levels of perfectionistic concerns and strivings may lead to either a “positive” or “harmful” experience of perfectionism (Stoeber and Otto, 2006). In the current study, perfectionism was assessed through the Short Almost Perfect Scale (SAPS; Rice et al., 2014), consisting of two subscales (each of four items) measuring the striving (high-performance expectations) and concern (self-critical performance evaluations) dimensions of perfectionism. The SAPS has demonstrated good psychometric properties in different countries (Rice et al., 2018), and its brevity facilitates its use in organizational research (e.g., Rice and Liu, 2020).

### The Relationship Between Perfectionism and Burnout

“Although initially applied only to service workers, burnout is now one of the most widely researched consequences of chronic and severe stress in employees in a wide range of different professions” (Childs and Stoeber, 2012, p. 347). Burnout has also been associated with a variety of workplace’s negative outcomes such as absenteeism, high turnover, low work morale, and reduced job performance (e.g., reduced quality of patient care), and its repercussion is evident also in marital and family problems (see Maslach et al., 2001; and Taris, 2006, for reviews). Antecedents of burnout have been widely studied, and recent meta-analytical studies in the work context have identified the following significant antecedents of burnout: influential demographic (e.g., Purvanova and Muros, 2010), situational (e.g., Alarcon, 2011), and personality factors (e.g., Swider and Zimmerman, 2010), including perfectionism (Childs and Stoeber, 2012). Given the degree of consensus among researchers on conceptualizing perfectionism through the two-factors model (i.e., strivings and concerns), the majority of studies has differentiated perfectionistic strivings and perfectionistic concerns when examining their relationships with burnout (e.g., Mitchelson and Burns, 1998; Childs and Stoeber, 2010, 2012; Taris et al., 2010; Caliskan et al., 2014). In this context, a number of studies found perfectionistic concerns to show positive relationships with burnout’s components [exhaustion, cynicism (or depersonalization), and inefficacy], whereas perfectionistic strivings showed no significant relationships (e.g., Mitchelson and Burns, 1998; Fairlie and Flett, 2003). This same pattern was confirmed by other studies that, in addition, detected negative correlations of perfectionistic strivings with the burnout’s inefficacy component (e.g., Caliskan et al., 2014; Li et al., 2014), while few studies found perfectionistic strivings to show positive correlations with burnout (Taris et al., 2010; Hrabluik et al., 2012). A meta-analysis conducted by Hill and Curran (2016) reported that perfectionistic strivings had minor negative or non-significant relationships with overall burnout and symptoms of burnout. On the contrary, perfectionistic concerns displayed medium-to-large and medium positive relationships with overall burnout and symptoms of burnout. A more recent meta-analysis carried out by Harari and colleagues (2018) indicated that the overall relationship between perfectionism and burnout was positive (σ = 0.21), as was the relationship between failure-avoiding perfectionism and burnout (σ = 0.34), whereas the relationship between excellence-seeking perfectionism and burnout was non-generalizable (σ = 0.08). In conclusion, findings indicate that only perfectionistic concerns consistently show positive relationships with burnouts.

Although in the sport and education domains research contributions advanced with several longitudinal contributions (e.g., Madigan et al., 2016), in the work domain research is all cross-sectional except for two longitudinal studies by Childs and Stoeber (2012) and Flaxman et al. (2012). In their study, Childs and Stoeber (2012) assessed two samples of employees: a sample of administrative and managerial staff working for the National Health Service and a sample of school teachers. They found in both samples that perfectionistic concerns were positively related to burnout and predicted increases in burnout over time. In another study, Flaxman et al. (2012) observed the relationships among perfectionism, cognitive vulnerabilities, and the outcomes of a respite from work, and they found a positive relationship between self-critical perfectionism and emotional exhaustion over time. Thus, despite cross-sectional analysis and meta-analysis reported perfectionistic concerns to be positively related to burnout and its components, while perfectionistic strivings seem to not be related to it, more longitudinal studies are needed to better understand the predictive role of perfectionism on burnout.

The most popular conceptualization of burnout was proposed by Maslach and Jackson (1981) who characterized it as a multidimensional phenomenon constituted by three core symptoms: depletion of emotional resources (emotional exhaustion); impersonal or cynical attitude (depersonalization or cynicism); and reduced personal competence, accomplishment, or efficacy (personal accomplishment or professional efficacy). The first symptom has been described as general feelings of being strained by demands being placed on the individual’s resources. The second core symptom is an interpersonal dimension of burnout that captures indifference or detachment from others. For the final symptom, lower levels of accomplishment or efficacy are indicative of higher levels of burnout. The most popular instrument to measure burnout comprehends these three dimensions and it is called the Maslach Burnout Inventory (MBI). However, MBI has been criticized on conceptual, practical, and psychometrical grounds, and Schaufeli et al. (2020) recently proposed a new conceptualization and measure of burnout, the Burnout Assessment Tool (BAT, Schaufeli et al., 2020), showing its cross-sectional validity within seven nationally representative samples (de Beer et al., 2020). The BAT conceptualizes burnout as a syndrome comprising four components—(1) exhaustion, (2) emotional impairment, (3) cognitive impairment, and (4) mental distance—following the distinction made by Schaufeli and Enzmann (1998), which considered these dimensions as burnout’s core symptoms with respect to other secondary, atypical ones. These four BAT dimensions are explained by the authors as follow: “(1) exhaustion, which refers to a severe loss of energy that results in feelings of both physical (tiredness, feeling weak) and mental (feeling drained and worn-out) exhaustion; (2) emotional impairment, which manifests itself in intense emotional reactions and feeling overwhelmed by one’s emotions; (3) cognitive impairment, which is cued by memory problems, attention and concentration deficits and poor cognitive performance; (4) mental distance, which signals the degree of being psychologically distancing from the work and by a strong reluctance or aversion toward it” (Schaufeli et al., 2019, p. 27). In addition to these core symptoms, burnout also includes secondary symptoms, such as: “psychological distress, which refers to non-physical symptoms resulting in psychological disorders, such as sleep problems, worries, feeling tense and anxious; psychosomatic complaints, which refers to physical complaints that cannot be explained by a physical disorder, but are exacerbated by or result from some psychological disorders; depressed mood that refers to a gloomy and sad mood and the inability to experience pleasure” (Schaufeli et al., 2020, p. 28).

BAT represents a relatively new measure of burnout, and, to our knowledge, there is a lack of studies addressing the relationship between perfectionism and burnout that adopt this new conceptualization. Thus, with the aim of contributing to the understanding of the role of the two-factor perfectionism model on this new conceptualization of burnout through a longitudinal perspective, we conducted a two-waves cross-lagged study. According to the results of the meta-analysis and previous longitudinal studies, the expected results are that perfectionistic concerns will positively relate to burnout and perfectionistic strivings will not according to the following hypotheses:

H1a: perfectionistic concerns at Time 1 will predict a relative increase of burnout at T2
H1b: perfectionistic strivings at Time 1 will not predict a relative increase of burnout at T2

## Method

### Participants

Participants were 191 workers (56% women). Most of them were employees without role responsibilities (63.9%), whereas the rest were managers (17.8%), freelancers (15.2%), and temporary workers (3.1%). The majority were employed in the private sector (58%), and their age ranged from 23 to 65 years (Mean = 42.5; St. Dev. = 11.75). In terms of their education, the majority of participants had a Bachelor’s or Master’s degree (55.5%), and the remaining had a high school (40.3%) and middle school (4.2%) diploma. Most of them were office workers (44.5%), and the remainder were teachers (14.7%), workmen (13.1%), freelancers (8.9%), doctors (7.9%), managers (4.2%), policemen (3.6%), and researchers (3.1%).

### Procedure

Data were collected through online versions of the BAT and SAPS questionnaires. Graduate students completing a course in Work and Organizational Psychology voluntarily assisted with data collection. They were asked to contact a limited number of available workers to participate in the study by sending them the link to the online questionnaire to be completed twice: in April and after 2 months (in June 2020). We decided to adopt this time-lag because the most rigid confinement due to the pandemic crisis in Italy was between March and the first days of May, and, thus, we managed to collect the data of the first questionnaire in the middle of this “first phase.” Then, after the first days of May, the restrictions were reduced and the Italians started the so-called “second phase” of the epidemiological crisis. Given the unpredictability of the pandemic trend, we thought that adopting a time-lag that allowed us to capture both phases was useful and interesting to study the impact of perfectionism on possible changes of burnout. Although at T1 respondents were 590, at T2 data were available only for 191 participants (32%)^1^. Participation was voluntary and not rewarded, and this might be the reason for the attrition rate. This study was conducted in line with the Helsinki Declaration (World Medical Association, 2001) as well as the Italian data protection regulation laws (Legislative Decree No. 196/2003). Participants signed informed consent and were debriefed on the aims of the research, confidentiality and anonymity issues, and instructed on how to fill the assessing questionnaires. Participants were made aware that they were free to withdraw from the study at any moment.

### Measures

#### Perfectionism

Perfectionism was measured using the SAPS (Rice et al., 2014), which taps each of the two essential elements of perfectionism exploiting standards (first subscale) as a measure of perfectionistic strivings, or high-performance expectations, and discrepancy (second subscale) as a measure of perfectionistic concerns, or perceived degree to which desired standards have been met. Sample items are, “I set very high standards for myself” (Standards) and “Doing my best never seems to be enough” (Discrepancy). Participants responded to SAPS items using a 5-point Likert scale ranging from 1 “strongly disagree” to 5 “strongly agree.”

#### Burnout

Burnout was measured using the BAT (BAT, Schaufeli et al., 2020) Italian version adapted by Consiglio et al. (unpublished)^2^. The 34 BAT items are distributed along six sub-dimensions that include the four core (four subscales) and secondary (two subscales) symptoms of burnout with the following items’ distribution: (1) exhaustion, eight items (item example: “When I get up in the morning, I lack the energy to start a new day at work”); (2) mental distance, five items (item example: “I feel indifferent about my job”); (3) emotional impairment, five items (item example “At work, I may overreact unintentionally”); (4) cognitive impairment, five items (item example: “At work, I struggle to think clearly”); (5) psychosomatic complaints, five items (item example “I suffer from palpitations or chest pain”); and (6) psychological distress, six items (item example “I have trouble falling or staying asleep.” Items were assessed on a 5-point Likert scale ranging from 1 “never” to 5 “always.”

### Data Analysis

Analyses were conducted with IBM SPSS Version 25 and AMOS Version 22. Zero-order correlations and Cronbach’s coefficients alpha were used to examine associations between variables and scales’ internal consistencies. To examine the relationships between the variables, a two-waves cross-lagged panel model (CLPM) was explored. This type of model is used to explore the structural relations of repeatedly measured constructs and to estimate the variables’ directional influence on each over time (Selig and Little, 2012).

Goodness of fit indices, such as the Comparative Fit Index (CFI), root mean square error of approximation (RMSEA), and standardized root mean residual (SRMR), for each model were checked (Bentler, 1990; McDonald and Marsh, 1990). In addition, chi-square (χ^2^) values were also reported for each model even though they were cautiously interpreted given the acknowledged chi-square’s sensitivity to sample sizes (Bentler and Bonnet, 1980; Hooper et al., 2008). CFI assesses the extent to which the tested model is superior to an alternative model in reproducing the observed covariance matrix (McDonald and Marsh, 1990). A cut-off criterion of CFI > 0.90 is needed to ensure that misspecified models are not accepted (Hooper et al., 2008). The RMSEA introduces a correction for lack of parsimony: a cut-off value close to 0.08 (Steiger, 2007) is accepted for an appropriate fit. The SRMR is an index of the averaged standardized residuals between the observed and hypothesized covariance matrices (Chen, 2007). SRMR values smaller than 0.08 are interpreted as a good fit (Hu and Bentler, 1999).

## Results

Table 1 shows the zero-order correlation among study variables and their reliability measured through Cronbach’s coefficient alpha. Interestingly, it seems that all the burnout sub-dimensions increased in Time 2, and thus a decrease in the psychological health might have occurred. Moreover, Gender was positively and significantly correlated to Perfectionistic Concerns at Time 1 and Psychological Distress and Psychosomatic complaints at both the two times, indicating that women had higher scores than men in these dimensions. Age appears to be negatively and significantly correlated to perfectionistic strivings and emotional exhaustion both a Time 1 and Time 2 and to mental distance and emotional impairment at Time 2.

**TABLE 1:** Descriptives and inter-correlations.

Variables	Mean	*SD*	1	2	3	4	5	6	7	8	9	10	11	12	13	14	15	16	17
1. Strivings T1	3.6	0.85	–																
2. Concerns T1	2.46	0.91	0.30**																
3. Strivings T2	3.56	0.88	0.67**	0.18**															
4. Concerns T2	2.52	0.91	0.21**	0.60**	0.26**														
5. Exhaustion T1	2.22	0.76	0.10	0.43**	0.09	0.31**													
6. Mental distance T1	1.64	0.68	–0.07	0.34**	–0.08	0.28**	0.53**												
7. Cognitive impairment T1	1.56	0.67	–0.07	0.40**	–0.07	0.25**	0.45**	0.58**											
8. Emotional impairment T1	1.70	0.72	0.02	0.35**	0.04	0.17*	0.44**	0.53**	0.64**										
9. Psychological stress T1	2.22	0.82	0.14	0.47**	0.13	0.31**	0.57**	0.36**	0.42**	0.54**									
10. Psychosomatic complaints T1	1.90	0.72	0.10	0.35**	0.11	0.16*	0.43	0.29	0.28	0.41	0.65**								
11. Exhaustion T2	2.29	0.82	0.12	0.36**	0.14**	0.50**	0.52**	0.42**	0.31**	0.35**	0.39**	0.32**							
12. Mental distance T2	1.81	0.79	0.03	0.31**	–0.06	0.45**	0.44**	0.66**	0.39**	0.35**	0.30**	0.22**	0.69**						
13. Cognitive impairment T2	1.72	0.76	0.05	0.39**	–0.04	0.54**	0.45**	0.52**	0.57**	0.40**	0.36**	0.23**	0.56**	0.75**					
14. Emotional impairment T2	1.80	0.80	0.03	0.36**	–0.01	0.44**	0.43**	0.51**	0.40**	0.54**	0.43**	0.28**	0.64**	0.72**	0.72**				
15. Psychological stress T2	2.29	0.91	0.11	0.41**	0.10	0.42**	0.50**	0.32**	0.36**	0.41**	0.70**	0.49**	0.68**	0.47**	0.46**	0.63**			
16. Psychosomatic complaints T2	2.07	0.81	0.03	0.34**	0.01	0.33**	0.40**	0.30**	0.24**	0.33**	0.51**	0.60**	0.60**	0.47**	0.38**	0.52**	0.74**		
17. Gender^*a*^	–	–	–0.10	0.18*	–0.06	0.15*	0.08	0.03	–0.04	0.05	0.15*	0.20**	0.11	0.03	0.03	0.10	0.15*	0.17*	
18. Age	42.5	11.74	−0.15*	–0.05	−0.22**	–0.13	−0.17*	–0.13	–0.10	–0.06	0.02	0.04	−0.18*	−0.15*	–0.12	−0.14*	–0.07	–0.03	–0.06

*p < 0.05, **p < 0.001.

**^*a*^Gender was codes as 1 = man, 2 = woman.**

The results of cross-lagged panel models are displayed in Table 2.

**TABLE 2:** Parameter estimates of the cross-lagged path model.

Paths	B	S.E.	CR
**Model 1 relationship between perfectionism and workaholism**			
Strivings T1 → Strivings T2	0.65**	0.05	11.84
Concerns T1 → Concerns T2	0.58**	0.07	8.72
Mental distance T1 → Mental distance T2	0.51**	0.04	11.56
Exhaustion T1 → Exhaustion T2	0.30**	0.05	5.86
Cognitive impairment → Cognitive impairment T2	0.49**	0.05	9.81
Emotional impairment → Emotional impairment T2	0.44**	0.05	9.27
Psychological distress → Psychological distress T2	0.58**	0.05	12.62
Psychosomatic complaints → Psychosomatic complaints T2	0.51**	0.05	9.77
Strivings T1 → Mental distance T2	0.02	0.05	0.27
Strivings T1 → Exhaustion T2	0.01	0.05	0.34
Strivings T1 → Cognitive impairment T2	0.02	0.05	0.30
Strivings T1 → Emotional impairment	–0.04	0.06	–0.77
Strivings T1 → Psychological distress	–0.01	0.06	–0.22
Strivings T1 → Psychosomatic complaints	–0.07	0.06	–1.34
Concerns T1 → Mental distance T2	0.14**	0.05	2.61
Concerns T1 → Exhaustion T2	0.22**	0.06	3.49
Concerns T1 → Cognitive impairment T2	0.17**	0.05	2.61
Concerns T1 → Emotional impairment T2	0.21**	0.06	3.62
Concerns T1 → Psychological distress	0.17**	0.06	2.85
Concerns T1 → Psychosomatic complaints	0.19**	0.06	3.32
Mental distance T1 → Strivings T2	–0.10	0.09	–1.04
Mental distance T1 → Concerns T2	0.15	0.10	1.47
Exhaustion T1 → Strivings T2	0.04	0.08	0.47
Exhaustion T1 → Concerns T2	0.04	0.09	0.44
Cognitive impairment T1 → Strivings T2	–0.06	0.10	–1.04
Cognitive impairment T1 → Concerns T2	0.01	0.11	0.06
Emotional impairment T1 → Strivings T2	0.07	0.09	0.72
Emotional impairment T1 → Concerns T2	–0.16	0.10	–1.51
Psychological distress T1 → Strivings T2	0.02	0.09	0.28
Psychological distress T1 → Concerns T2	0.12	0.10	1.23
Psychosomatic complaints → Strivings T2	0.04	0.09	0.42
Psychosomatic complaints → Concerns T2	–0.14	0.10	–1.42

**p < 0.001. T1, Time 1; T2, Time 2; B, unstandardized coefficient; S.E., standard error of the estimate; CR, critical ratio.

Goodness of fit of the proposed cross-lagged path model was: χ^2^ (44, *N* = 191) = 126.674, *p* < 0.0001; RMSEA = 0.10; CFI = 0.96; SRMR = 0.07. CFI value was excellent, and SRMR was acceptable, whereas RMSEA seemed to be unsatisfactory. However, Kenny et al. (2015) pointed out that with small degrees of freedom, the RMSEA too often falsely indicates a poorly fitting model. In general, it seems that with samples < 500, RMSEA might incorrectly suggest that models do not fit closely. Thus, we mainly relied on the CFI and SRMR.

All the stability and cross-lagged paths coefficients are presented in Table 2. Statistically significant effects emerged from Perfectionistic Concern at T1 to Mental Distance at T2 (*B* = 0.14, *p* < 0.001), Exhaustion at T2 (*B* = 0.22, *p* < 0.001), Cognitive Impairment at T2 (*B* = 0.17, *p* = 0.01), Emotional Impairment at T2 (*B* = 0.21, *p* < 0.001), Psychological Distress at T2 (*B* = 0.17, *p* < 0.001), and Psychosomatic complaints (*B* = 0.19, *p* < 0.001). As expected, the effects of Perfectionistic Strivings at T1 on all the Burnout dimensions were not significant. All the burnout and perfectionism dimensions were stable across time. The reciprocal effects of all the burnout’s dimension at T1 and perfectionism’s dimension at T2 were not significant.

The above-reported data support previous studies’ results relating only concerns and not perfectionistic strivings to the core and secondary symptoms of burnout’s dimensions. This remains true even with particularly stressing workplace conditions such as those brought about by the COVID emergency, suggesting that maladaptive aspects of perfectionism are not smoothed or (maybe) enhanced by crisis situations and remain stable over time. In addition, the data show that BAT predictions are similarly valid and informative to MBI predictions in longitudinally relating perfectionism to burnout.

## Discussion

This study investigated the role of perfectionistic strivings and concerns in predicting burnout over time. In line with previous longitudinal (Childs and Stoeber, 2012) and meta-analytic studies (Hill and Curran, 2016; Harari et al., 2018), results indicated that perfectionistic concerns predicted all the burnout dimensions. Thus, there seems to be converging evidence that perfectionistic concerns, and not perfectionistic strivings, play a crucial role in the experience of burnout. It seems plausible that the self-defeating aspects of excessive self-criticisms, such as regularly evaluating the self’s performance to reach standards as inadequate, capture self-evaluative tendencies that render individuals vulnerable to the accrual of stress (Hill and Curran, 2016). This has been made evident in previous research underlining the association between perfectionistic concerns, threat appraisals, anxiety, and avoidant coping (e.g., Rice et al., 2006; Stoeber and Rennert, 2008; Hill et al., 2010). Thus, according to Swider and Zimmerman (2010), perfectionistic concerns may be a crucial personality factor rendering individuals prone to burnout (Swider and Zimmerman, 2010). Results, showing that perfectionistic strivings are not predictive of burnout strengthen the assumption that the two perfectionism dimensions are related to an adaptive (perfectionistic strivings) and maladaptive form of perfectionism (perfectionistic concerns). All in all, perfectionism might represent a crucial personality variable in the pandemic crises since it can be also related to obsessive-compulsive traits (e.g., Pinto et al., 2017), which are increasing and seems to appear as a way to deal with anxiety and fear of being infected (Kenny et al., 2015).

Results from our study should be considered in light of some limitations. One limitation concerns the convenience sampling procedures, which introduces potential biases when interpreting our results. In fact, the workers’ sample involved is unlikely to be representative of all workers’ population, undermining the possibility of making generalizations.

Second, because this study used self-reported measures, the collected data may be affected by participants’ acquiescence and needs for social desirability. Future studies could improve on the current study’s design by using objective measures, such as stress biomarkers (like salivary cortisol and α−amylase). Finally, as suggested by Burisch (2002) the relatively short time lag used in this study was opted in order to avoid attrition among participants and capture the specific COVID-19 crisis’s proximal outcomes. Future studies should consider longer time lags to investigate the stability over longer time periods of the collected measures.

Despite these limitations, the current study extends and enhances the current knowledge on which perfectionism dimensions predict burnout in work contexts. Our findings, in line with previous studies, showed that only perfectionistic concerns are significantly and positively related to the burnout dimensions. Thus, clinicians and employers should monitor the presence of these maladaptive perfectionistic attitudes that may cause individual’s burnout, and exploit this information to design and implement programs devoted to smooth employee’s perfectionistic traits to improve their well-being (Clark et al., 2010, 2014). As reported by Harari et al. (2018), managers should make attempts to supervise employees high on perfectionism and, if needed, adopt interactional procedures that may mitigate their tendencies toward perfectionistic concerns. In general, organizational interventions should promote workers’ sensibilization of the detrimental outcomes of perfectionistic concerns.

## Data Availability Statement

The raw data supporting the conclusions of this article will be made available by the authors, without undue reservation.

## Ethics Statement

Ethical review and approval was not required for the study on human participants in accordance with the local legislation and institutional requirements. The patients/participants provided their written informed consent to participate in this study.

## Author Contributions

PS contributed to the research design, data collection, data analysis, and write up. LK contributed to the research design and data analysis. CB contributed to the research design and data collection. AE and GC contributed to the write up. All authors contributed to the article and approved the submitted version.

## Conflict of Interest

The authors declare that the research was conducted in the absence of any commercial or financial relationships that could be construed as a potential conflict of interest.

## Footnotes

1 Attrition analysis was conducted for examining the differences between the participants who completed the two waves and participants who dropout after completing the first questionnaire. Results of the ANOVA analysis comparing the means of the study’s variables on the two targets of participants reported no differences for perfectionistic standard, emotional exhaustion, and psychological distress, whereas differences were found for the other burnout dimensions and perfectionistic concerns in the way that participants who dropped out reported higher means in those variables. Although these differences should be taken into account, the particular timing and context where the study is collocated could have had an impact on their dropout. In fact, differences were detected also in the participants of the two-waves sample (*N* = 190). Comparing their answers in the two time points, results showed that participants reported higher score at the second questionnaire for some of the burnout variables, such as mental detachment, cognitive impairment, emotions impairment, and psychosomatic complaints. It is reasonable to suppose this trend could be due to the pandemic crisis situation, which could have had a role in enhancing the level of this dimensions.

2 Since the BAT is a new measure, and the validation article for the Italian version is not published yet, a confirmatory factorial analysis (CFA) was conducted using SEM to confirm the six-correlated factor model used in this study. Results of the CFA indicated that model fit the data very well both in the sample at Time 1 (*N* = 590; *X*^2^ = 1593.171, *df* = 512; SRMR = 0.057; CFI = 0.91; RMSEA = 0.06) and in the two-wave sample at T2 (*N* = 190; *X*^2^ = 1048.150, *df* = 512; SRMR = 0.062; CFI = 0.89; RMSEA = 0.07). For completeness, we also tested the second-order model of burnout on the sample at T1 and results are consistent to Schaufeli et al. (2020) showing that the fit of the model was adequate (*N* = 590; *X*^2^ = 1828.559, *df* = 521; SRMR = 0.072; CFI = 0.89; RMSEA = 0.06), although it was slightly worse than the six-factor correlated model.

## References

[B1] Alarcon G. M. (2011). A meta-analysis of burnout with job demands, resources, and attitudes. *J. Voc. Behav.* 79 549–562. 10.1016/j.jvb.2011.03.007

[B2] Bentler P. M. (1990). Comparative fit indexes in structural models. *Psychol. Bull.* 107, 238–246. 10.1037/0033-2909.107.2.238 2320703

[B3] Bentler P. M. Bonnet D. C. (1980). Significance tests and goodness of fit in the analysis of covariance structures. *Psych. Bull.* 88 588–606. 10.1037/0033-2909.88.3.588

[B4] Burisch M. (2002). A longitudinal study of burnout: the relative importance of dispositions and experiences. *Work Stress* 16 1–17. 10.1080/02678370110112506

[B5] Caliskan S. C. Arikan S. C. Saatci E. Y. (2014). SMEs context of Turkey from the relational perspective of members’ perfectionism, work family conflict and burnout. *Int. J. Bus. Soc. Sci.* 5 129–139.

[B6] Chen F. F. (2007). Sensitivity of goodness of fit indexes to lack of measurement invariance. *Struct. Eq. Mod. Mult. J.* 14 464–504. 10.1080/10705510701301834

[B7] Childs J. H. Stoeber J. (2010). Self-oriented, other-oriented, and socially prescribed perfectionism in employees: relationships with burnout and engagement. *J. Work. Beh. Health* 25 269–281. 10.1080/15555240.2010.518486

[B8] Childs J. H. Stoeber J. (2012). Do you want me to be perfect? two longitudinal 20studies on socially prescribed perfectionism, stress and burnout in the workplace. *Work Stress.* 26 347–364. 10.1080/02678373.2012.737547

[B9] Clark M. A. Lelchook A. M. Taylor M. L. (2010). Beyond the big five: how narcissism, perfectionism, and dispositional affect relate to workaholism. *Pers. Indiv. Diff.* 48 786–791. 10.1016/j.paid.2010.01.013

[B10] Clark M. A. Michel J. S. Zhdanova L. Pui S. Y. Baltes B. B. (2014). All work and no play? a meta-analytic examination of the correlates and outcomes of workaholism. *J. Manag.* 42 1836–1873. 10.1177/0149206314522301

[B11] de Beer L. T. Schaufeli W. B. De Witte H. Hakanen J. J. Shimazu A. Glaser J. (2020). Measurement invariance of the Burnout Assessment Tool (BAT) across seven cross-national representative samples. *Int J Environ Res Public Health* 17:5604. 10.3390/ijerph17155604 32756483 PMC7432716

[B12] Fairlie P. Flett G. L. (2003). “Perfectionism at work: Impacts on burnout, job satisfaction, and depression,” in *Poster Presented at the 111th Annual Convention of the.* (Toronto, Ont: American Psychological Association).

[B13] Falco A. Dal Corso L. Girardi D. De Carlo A. Barbieri B. Boatto T. (2017). Why is perfectionism a risk factor for workaholism? the mediating role of irrational beliefs at work. *TPM.* 24 583–600. 10.4473/tpm24.4.8

[B14] Flaxman P. E. Ménard J. Bond F. W. Kinman G. (2012). Academics’ experiences 1of a respite from work: effects of self-critical perfectionism and perseverative cognition on post respite well-being. *J. App. Psych.* 97 854–865. 10.1037/a0028055 22545621

[B15] Harari D. Swider B. W. Steed L. B. Breidenthal A. P. (2018). Is perfect good? a meta-analysis of perfectionism in the workplace. *J. App Psych.* 103 1121–1144. 10.1037/apl0000324.supp 29927262

[B16] Hill A. P. Curran T. (2016). Multidimensional perfectionism and burnout: a meta-analysis. *Pers. Soc. Psych. Rev.* 20 269–288. 10.1177/1088868315596286 26231736

[B17] Hill A. P. Hall H. K. Appleton P. R. Murray J. J. (2010). Perfectionism and 19burnout in canoe polo and kayak slalom athletes: the mediating influence of validation and 20growth-seeking. *Sport Psych.* 24 16–34. 10.1123/TSP.24.1.16

[B18] Hooper D. Coughlan J. Mullen M. R. (2008). Structural equation modelling: guidelines for determining model fit. *Elect. J. Bus. Res. Meth.* 6 53–60. 10.21427/D7CF7R

[B19] Hrabluik C. Latham G. P. McCarthy J. M. (2012). Does goal setting have a dark side? the relationship between perfectionism and maximum versus typical employee performance. *Int. Pub. Manag. J.* 15, 5–38. 10.1080/10967494.2012.684010

[B20] Hu L. T. Bentler P. M. (1999). Cutoff criteria for fit indexes in covariance structure analysis: conventional criteria versus new alternatives. *Struc. Equat. Modl. Mult. J.* 6 1–55. 10.1080/10705519909540118

[B21] Ji G. Wei W. Yue K. C. Li H. Shi L. J. Ma J. D. (2020). Effects of the COVID-19 pandemic on obsessive-compulsive symptoms among university students: prospective cohort survey study. *J. Med. Int. Res.* 22:e21915. 10.2196/21915 32931444 PMC7528732

[B22] Kenny D. A. Kaniskan B. McCoach D. B. (2015). The performance of RMSEA in models with small degrees of freedom. *Sociol. Methods Res.* 44, 486–507. 10.1177/0049124114543236

[B23] Li X. Hou Z.-J. Chi H.-Y. Liu J. Hager M. J. (2014). The mediating role of coping in the relationship between subtypes of perfectionism and job burnout: a test of the 2 × 2 model of perfectionism with employees in China. *Pers. Indiv. Diff.* 58 65–70. 10.1016/j.paid.2013.10.007

[B24] Madigan D. J. Stoeber J. Passfield L. (2016). Motivation mediates the perfectionism–burnout relationship: a three-wave longitudinal study with junior athletes. *J. Sport Ex. Psych.* 38 341–354. 10.1123/jsep.2015-0238 27383053

[B25] Maslach C. (2003). Job Burnout: new directions in research and intervention. *Curr. Dir. Psych. Sci.* 12 189–192. 10.1111/1467-8721.01258

[B26] Maslach C. Jackson S. E. (1981). The measurement of experienced burnout. *J. Occup. Behav.* 2 99–113. 10.1002/job.4030020205

[B27] Maslach C. Schaufeli W. B. Leiter M. P. (2001). Job Burnout. *Ann. Rev. Psych.* 52 397–422. 10.1146/annurev.psych.52.1.397 11148311

[B28] McDonald R. P. Marsh H. W. (1990). Choosing a multivariate model: noncentrality and goodness of fit. *Psych. Bull.* 107 247–255. 10.1037/0033-2909.107.2.247

[B29] Mitchelson J. K. Burns L. R. (1998). Career mothers and perfectionism: stress at work and at home. *Pers. Indiv. Diff.* 23 477–485. 10.1016/S0191-8869(98)00069-5

[B30] Pinto A. Dargani N. Wheaton M. G. Cervoni C. Rees C. S. Egan S. J. (2017). Perfectionism in obsessive-compulsive disorder and related disorders: what should treating clinicians know? *J. Obs. Comp. Rel. Disord.* 12 102–108. 10.1016/j.jocrd.2017.01.001

[B31] Purvanova R. K. Muros J. P. (2010). Gender differences in burnout: a meta-21analysis. *J. Voc. Behav.* 77 168–185. 10.1016/J.JVB.2010.04.006

[B32] Rice K. G. Liu Y. (2020). Perfectionism and burnout in R&D teams. *J. Couns. Psych.* 67 303–314. 10.1037/cou0000402 31855020

[B33] Rice K. G. Richardson C. M. Tueller S. (2014). The short form of the revised almost perfect scale. *J. Pers. Assess.* 96 368–379. 10.1080/00223891.2013.838172 24090303

[B34] Rice K. G. Vergara D. T. Aldea M. A. (2006). Cognitive-affective mediators of perfectionism and college student adjustment. *Pers. Indiv. Diff.* 40 463–6473. 10.1016/j.paid.2005.05.011

[B35] Rice S. P. Loscalzo Y. Giannini M. Rice K. G. (2018). Perfectionism in Italy and the USA: measurement invariance and implications for cross-cultural assessment. *Europ. J. Psych. Assess.* 36 207–211. 10.1027/1015-5759/a000476

[B36] Schaufeli W. B. Enzmann D. (1998). *The Burnout Companion to Study & Practice: A CriticalAnalysis*. Philadelphia: Taylor & Francis.

[B37] Schaufeli W. B. De Witte H. Desart S. (2019). *Manual Burnout Assessment Tool (BAT).* Leuven: KU. 10.3390/ijerph17249495 PMC7766078 33352940

[B38] Schaufeli W. B. Desart S. De Witte H. (2020). Burnout Assessment Tool (BAT)—development, validity, and reliability. *Int. J. Environ. Res. Public Health* 17:9495. 10.3390/ijerph17249495 33352940 PMC7766078

[B39] Selig J. P. Little T. D. (2012). “Autoregressive and cross-lagged panel analysis for longitudinal data,” in *Handbook of developmental research methods*, eds Laursen B. Little T. D. Card N. A. (New York: Guilford Press), 265–278.

[B40] Slaney R. B. Rice K. G. Mobley M. Trippi J. Ashby J. S. (2001). The revised almost perfect scale. *Measur. Eval. Couns. Dev.* 34 130–145. 10.1080/07481756.2002.12069030

[B41] Steiger J. H. (2007). Understanding the limitations of global fit assessment in structural equation modeling. *Pers. Indiv. Diff.* 42 893–898. 10.1016/j.paid.2006.09.017

[B42] Stoeber J. Otto K. (2006). Positive conceptions of perfectionism: approaches, evidence, challenges. *Pers. Soc. Psych. Rev.* 10 295–319. 10.1207/s15327957pspr1004_2 17201590

[B43] Stoeber J. Rennert D. (2008). Perfectionism in school teachers: relations with stress appraisals, coping styles, and burnout. *Anx Stress Cop.* 21 37–53. 10.1080/10615800701742461 18027123

[B44] Stoeber J. Stoeber F. S. (2009). Domains of perfectionism: prevalence and relationships with perfectionism, gender, age, and satisfaction with life. *Pers. Indiv. Diff.* 46 530–535. 10.1016/j.paid.2008.12.006

[B45] Swider B. W. Zimmerman R. D. (2010). Born to burnout: a meta-analytic path model of personality, job burnout, and work outcomes. *J. Voc. Behav.* 76 487–506. 10.1016/j.jvb.2010.01.003

[B46] Taris W. T. (2006). Is there a relationship between burnout and objective performance? a critical review of 16 studies. *Work Stress* 20 316–334. 10.1080/02678370601065893

[B47] Taris T. W. Van Beek I. Schaufeli W. B. (2010). Why do perfectionists have ahigher burnout risk than others? A mediational analysis. *Rom. J. Appl. Psychol.* 12, 1–7

[B48] World Medical Association (2001). World medical association declaration of helsinki: ethical principles for medical research involving human subjects. *Bull. World Health Organ.* 79, 373–374. 11357217 PMC2566407

